# A Multidisciplinary Approach for the Assessment of Origin, Fate and Ecotoxicity of Metal(loid)s from Legacy Coal Mine Tailings

**DOI:** 10.3390/toxics9070164

**Published:** 2021-07-10

**Authors:** Honorine Gauthier-Manuel, Diane Radola, Flavien Choulet, Martine Buatier, Raphaël Vauthier, Tatiana Morvan, Walter Chavanne, Frédéric Gimbert

**Affiliations:** 1 UMR CNRS 6249 Chrono-Environnement, Université Bourgogne Franche-Comté, 16 Route de Gray, CEDEX, 25030 Besançon, France; honorine.gauthier-manuel@univ-fcomte.fr (H.G.-M.); diane5795@yahoo.fr (D.R.); flavien.choulet@univ-fcomte.fr (F.C.); martine.buatier@univ-fcomte.fr (M.B.); 2 Conservatoire d’Espaces Naturels de Franche-Comté, Maison de l’environnement de Bourgogne Franche-Comté, 7 rue Voirin, 25000 Besançon, France; raphael.vauthier@cen-franchecomte.org (R.V.); tatiana.morvan@cen-franchecomte.org (T.M.); 3 Granulats De Franche Comté, 10 rue de Franche-Comté-Bât C, 25480 Ecole-Valentin, France; walter.chavanne@eqiom.com

**Keywords:** coal mineralogy, acid mine drainage, biomonitoring, bioavailability, *Chironomus riparius*

## Abstract

Over the course of history, the development of human societies implied the exploitation of mineral resources which generated huge amounts of mining wastes leading to substantial environmental contamination by various metal(loid)s. This is especially the case of coal mine tailings which, subjected to weathering reactions, produce acid mine drainage (AMD), a recurring ecological issue related to current and past mining activities. In this study, we aimed to determine the origin, the fate and the ecotoxicity of metal(loid)s leached from a historical coal tailing heap to the Beuveroux river (Franche-Comté, France) using a combination of mineralogical, chemical and biological approaches. In the constitutive materials of the tailings, we identified galena, tetrahedrite and bournonite as metal-rich minerals and their weathering has led to massive contamination of the water and suspended particles of the river bordering the heap. The ecotoxicity of the AMD has been assessed using *Chironomus riparius* larvae encaged in the field during a one-month biomonitoring campaign. The larvae showed lethal and sub-lethal (growth and emergence inhibition and delay) impairments at the AMD tributary and near downstream stations. Metal bioaccumulation and subcellular fractionation in the larvae tissues revealed a strong bioavailability of, notably, As, Pb and Tl explaining the observed biological responses. Thus, more than 70 years after the end of mining operations, the coal tailings remain a chronic source of contamination and environmental risks in AMD effluent receiving waters.

## 1. Introduction

The exploitation of mineral resources has contributed to the socio-economic development of human populations over the last millennia [1,2]. The mineral related activities also constitute the most ancient and significant source of waste production. These remains, often rich in metallic elements, may represent an environmental risk during their production but also after the end of the operations. Indeed, once abandoned they can be subjected to weathering phenomenon and subsequently release metal(loid)s in various environmental compartments [3]. This is especially the case of sulfidic residues which, exposed to air and water, generate acidic conditions favoring the leaching of metals that originate from native rocks of the tailings [4]. This process is well-known as acid mine drainage (AMD).

In the context of AMD, aquatic ecosystems are particularly vulnerable since they are the first receiving compartments of such effluents. Indeed, AMD waters can on one hand, infiltrate and reach groundwaters [5] and, on the other hand, directly flow into the surface hydrological network [6]. As a consequence, environmental monitoring and ecological risk assessment of water bodies potentially affected by AMD effluents are recurring ecological issues worldwide. It is nowadays well admitted that a single chemical analysis of total metal concentrations in water or sediment may be insufficient for environmental risk assessment purposes [7]. Biological approaches are required for an accurate assessment of the bioavailability and potential toxicity of metal(loid)s present in exposure matrices [8]. To this aim, passive and active biomonitoring approaches have been developed during the last decades. The first one uses local (i.e., indigenous) populations to measure the impact of contaminants in the environment. However, local populations can develop adaptative traits through generations particularly in context of past (i.e., long term) pollutions [9,10]. Hence, active biomonitoring, by exposing laboratory-reared animals in the field, is an interesting alternative for a relevant environmental quality assessment. 

There is a large panel of bioindicators available through numerous taxa and trophic levels [11,12]. Among aquatic macroinvertebrates, chironomids fulfil criteria to be considered as a good bioindicator of water quality: ubiquity, low mobility and abundance (until 50,000 larvae per m²), key role and trophic position in freshwater ecosystems, ability to bioaccumulate metals with a certain tolerance [13]. Moreover, several active biomonitoring devices have been recently developed with chironomid larvae allowing the measurement of life traits such as survival, growth or emergence in the field [14,15]. Complementarily, the analysis of bioaccumulation in chironomid tissues allows to acquire further in the risk assessment by considering metal bioavailability and transfer into sensitive internal compartments [16]. The bioavailability is a key concept in ecotoxicology including three levels: environmental availability, environmental bioavailability and toxicological availability [17,18]. The environmental availability implies physicochemical processes driving the distribution of contaminants between the solid and liquid phases of the exposure matrix. This aspect is classically assessed using chemical approaches (total or extractable concentrations). The environmental bioavailability refers to specific physiological processes which condition the bioaccumulation levels of the contaminant according to uptake, storage and excretion mechanisms of the studied species. Finally, the toxicological bioavailability designates the portion of assimilated chemical that reaches and interacts with the sites of toxic action. The consideration of bioavailability combined with in situ approaches as active biomonitoring, provides relevant environmental diagnoses [15]. 

Therefore, the main objective of this study is to assess the potential current environmental impacts linked to the presence of legacy coal tailings in a former mining district where a recent hydrogeological survey pointed out contaminations of surface waters by various metal(loid)s. Beyond a case study, we proposed here an original multidisciplinary approach carried out to investigate the origin, fate and ecotoxicity of metal(loid)s in a potential AMD. From an operational point of view, we intended: (i) to characterize the main metal-bearing phases notably through a mineralogical study of constitutive materials of the tailing; (ii) to determine the fate of the metallic elements in the overlying waters and suspended particles of the stream bordering the heap; and (iii) to assess the toxicological bioavailability of metal(loid)s through active biomonitoring approaches using *Chironomus riparius* larvae.

## 2. Materials and Methods

### 2.1. Study Site 

**Figure 1:**
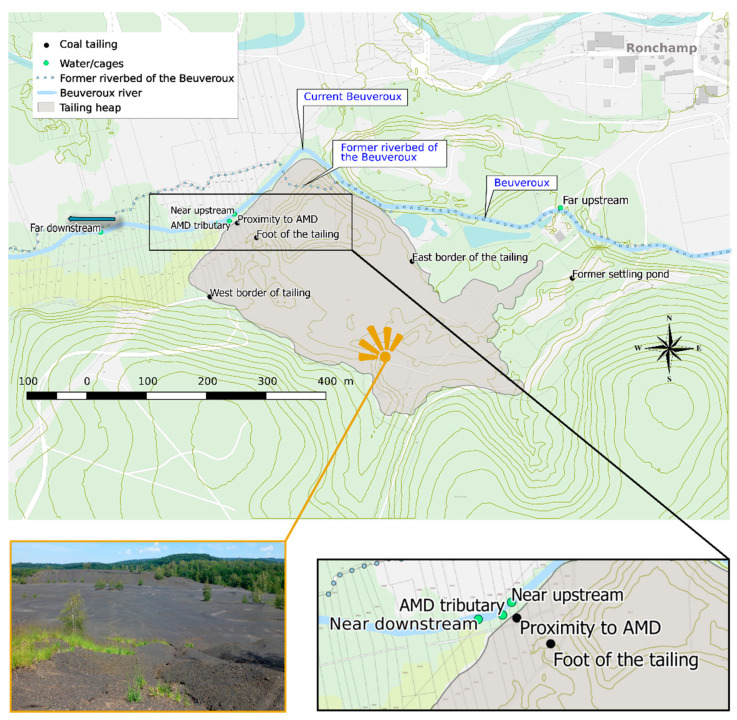
Map of the study area, presenting the coal tailing sampling stations over the heap (black points) and the water sampling/caging stations in the Beuveroux river (turquoise points).

The study site is located in Eastern France in the Burgundy Franche-Comté region within the coal basin of Ronchamp (Northern Haute-Saône). For more than 200 years (from the 18th to the middle of the 20th century), this oil shale-rich basin has been exploited for coal production and the mining wastes scattered around the shafts and galleries in the outcrop areas [19,20]. The most important deposit site is located in the municipality of Magny-Danigon, where the waste heap called “du Triage” covers an area of 17 ha and represents a volume of almost one million tons. At the foot of the north face of the heap flows a small river, the Beuveroux (Figure 1). In the early 80’s, the tailing has known some reworkings (remobilization and sorting) to extract the remains of coal. These operations caused a displacement of the heap and a covering of a part of the Beuveroux by residual materials leading to a modification of its riverbed (Figure 1). Since 2011, the environmental management of the site has been entrusted to the Conservatoire d’Espaces Naturels de Franche-Comté (CEN Franche-Comté) which monitors species of high conservation value (e.g., plants such as *Drosera rotundifolia* or dragonflies such as *Ischnura pumilio*) in four ponds dug on the surface of the heap.

### 2.2. Sampling Strategy

#### 2.2.1. Coal Tailing Sampling from the Heap

Samples of shale residue materials (*n* = 5) were hand collected on the surface in different areas of the waste dump in order to consider their potential spatial heterogeneity (structure, composition) related to different extraction processes (sorting area, settling pond…) (Figure 1). The samples were dried at room temperature and stored for further analyses. 

#### 2.2.2. Water and Suspended Particles Sampling

Five stations have been chosen along the Beuveroux river. The choice of the station locations was supported by the identification of a tributary, fed by a resurgence discovered at the foot of the waste dump (North-West part) and which could be considered as a potential AMD (Figure 1). Two stations were set up upstream the confluence between this AMD tributary and the Beuveroux, and two others downstream (Figure 1). The localization of the stations at increasing distances (near and far) from this AMD has for purpose to study its influence on the water quality along the Beuveroux. At each station, water was sampled (*n* = 3) in 50 mL tubes at 30 cm depth, filtered at 0.45 µm, then acidified with 0.5% HCl and stored at 5 °C before analysis. A particle trap was also deployed at each station for seven days. After their retrieval, particles were freeze-dried and stored at room temperature before analysis.

### 2.3. Analyses of Materials from the Coal Waste Dump

#### 2.3.1. Mineralogical Characterization

In order to identify the crystalline phases, mineralogical composition of shale samples was obtained by X-Ray Diffraction (XRD) using a D8 Advance Brucker diffractometer equipped with a LinxEye detector (CuKa radiation at 1.54184 A, 40 kV/40 mA) hosted at the Utinam Institute (University Bourgogne Franche-Comte, France). Powdered (<50 µm) bulk material samples were treated between 3 to 60° (2ϴ divergent slit) with a step-scan of 0.019° (2θ) and 1 s/step. The EVA software package was used for data processing and phase identification.

To investigate the main metal-bearing minerals, samples of shale residues were cut to prepare 30 µm-thick polished thin sections. Optical investigations using a FEI Quanta 450 scanning electron microscopy (SEM) in back-scattered electron mode (BSE, acceleration voltage at 15 kV, low vacuum mode between 0.7 and 0.8 mbar) were carried out at Femto-ST Institute (University Bourgogne Franche-Comte, France). Observations were complemented by semi-quantitative analysis using energy-dispersive spectrometry (EDS) and Edax Genesis software package.

#### 2.3.2. Elemental Composition

Materials (oil shale residues) were grounded, sieved to 80 µm and completely digested (m = 0.250 g) in PTFE vials on hot plates in 5 mL of fluorhydric acid (47% HF) and 1.5 mL of perchloric acid (65% HClO_4_) according to [21]. The mixture was allowed to evaporate to near complete dryness, and the residues were dissolved in diluted HNO_3_ and filtered at 1µm for analysis by atomic emission spectroscopy (ICP AES, iCAP 6000 Series, Thermo Scientific). The reliability of analytical results was evaluated with a certified reference soil (Standard Reference Material^®^ 2709a) with an average recovery efficiency of 98 ± 26% for all the metal(loid)s analysed.

### 2.4. Water and Suspended Particle Analyses

#### 2.4.1. Physico-chemical Analyses of Water

During field sessions, pH, conductivity, dissolved oxygen and temperature were regularly measured in waters with a multiparameter probe (WTW, Multi 3420). Water samples were also analyzed for their metal(loid) dissolved concentrations using ICP-MS (XSeries 2, Thermo Scientific). Precision and accuracy of analyses were evaluated with a certified water (European Reference Material ERM^®^ CA022a) with an average recovery efficiency of 100 ± 7%.

#### 2.4.2. Suspended Particle Contamination

Metal(loid) contents were determined in suspended particles, after hot digestion (0.5 g) in aqua regia (HNO_3_/HCl, 2:5 *v/v*, Sigma-Aldrich, purity 99.9%), by ICP-MS. Analytical precision and accuracy were evaluated with a certified reference material (calcareous loam soil n°141R). The average recovery efficiency was 107 ± 24%.

### 2.5. Ecotoxicological Assessment

#### 2.5.1. Obtaining Chironomus Rirarius Larvae

*Chironomus riparius* (*Hexapoda*; *Diptera*) larvae were bred in the lab under controlled conditions (temperature = 20 °C, constant oxygenation, feeding *ad libitum* with organic oat flour) according to standardized methods [22]. In order to obtain fourth instar larvae for experimentations, egg masses were collected in the breeding and left to hatch (2–3 days) at room temperature in 20 mL pillboxes filled with breeding water (40% dechlorinated tap water and 60% ultrapure (UP) water) (20 °C, 250 μS.cm^−1^, pH = 7.5, 16:8 h light:dark photoperiod). Synchronized new born organisms (first instar larvae) were then transferred to 3 L containers filled with 1.5 L breeding water and a thin layer of sand, and were fed *ad libitum* each day with 1 mg organic oat flour.larvae^−1^.day^−1^ [23]. After two to three days, second instar larvae were individually sorted and 20 individuals were placed in 0.6 L beakers, filled with 0.1 L clean sand and 0.4 L breeding water, and fed as previously. After four to five days, larvae reached the fourth instar and were ready to be exposed in the field.

#### 2.5.2. Active Biomonitoring Campaign

**Figure 2:**
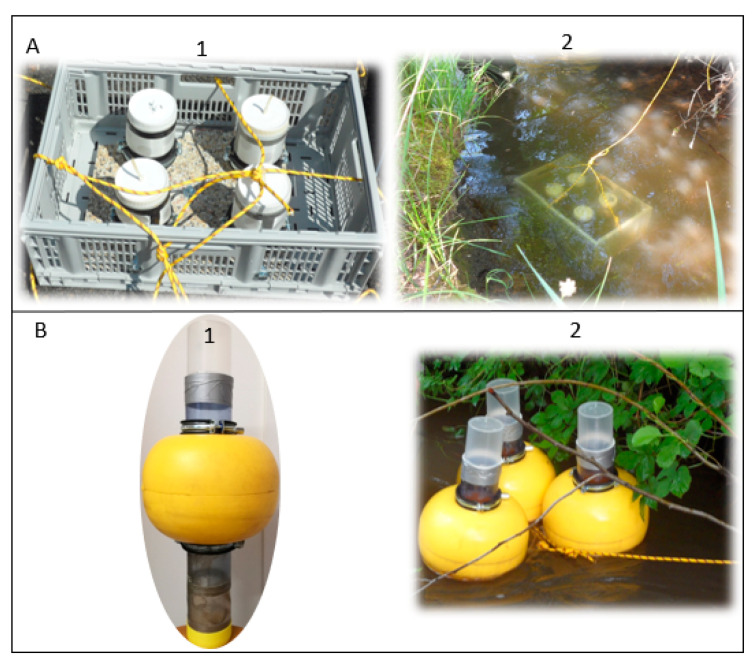
Photographs of active biomonitoring devices dedicated to in situ exposure of *Chironomus riparius* larvae. (**A**): submerged cages dedicated to the assessment of growth, survival and bioaccumulation; (**B**): Floating cages dedicated to the monitoring of emergence; 1: before deployment and 2: installed devices.

Two types of devices were designed and constructed before their deployment in the five field stations (Figure 2). 

The first device aimed at measuring mortality, growth and bioaccumulation (after 7 days of exposure) (Figure 2A). It is a crate weighted with a concrete slab at its bottom. It contained cages in triplicate and a suspended particles trap fixed to the crate via stainless steel mounting brackets (Figure 2A). On the side of cages, holes were drilled and covered with nylon mesh (500 µm) to let water and suspended particles circulate inside devices. A layer of 2 cm of non-contaminated sediment was deposited at the bottom of the cages and 20 fourth instar chironomid larvae were introduced before their disposal in the river. The crates were moored on the bank with a rope. After 7 days, cages were removed, brought back to the lab where larvae were retrieved by gently sieving of the sand, pooled per replicate (*n* = 3) in cryotubes and sacrificed by freezing at −80 °C.

The second caging device was dedicated to the monitoring of the emergence of adults (over 30 days of exposure) (Figure 2B). It corresponded to floating cages (Figure 2B). A PVC tube (Ø = 90 mm, H = 50 cm) was fenestrated at its bottom end and the holes covered by 500 µm nylon mesh (allowing for water and suspended particles circulation in the cage). Two protective caps (90 and 77 mm diameters) were nested to create an exposure chamber for chironomids at the bottom end of the cage. We filled this exposure chamber with a 2 cm layer of silica sand and 20 fourth instar larvae. At the top of the cage, a transparent plastic box (Ø = 90 mm, H = 10 cm) was fixed with a screw cap, allowing retrieval of adults. Two to three times a week, the cages were inspected and the emerged individuals were counted, collected and the gender identified. At each station, these floating cages were deployed in triplicate.

#### 2.5.3. Tissue Fractionation and Metal Analyses

We separated the soluble and insoluble internal fractions of larval tissues through a simplified subcellular fractionation procedure [15]. The soluble fraction corresponds to the cytosolic compartment and represents the metal fraction truly bioavailable to larvae in terms of individual toxicity (i.e., toxicological bioavailability; [16,24]) and potentially transferable to the food web (trophically bioavailable fraction; [25]). On the other hand, the insoluble fraction contains the gut content and non-sensitive compartments such as the exoskeleton, granules and cellular debris [16].

Briefly, partially thawed larvae were homogenized (Ultra-Turax) in 3 mL of ice-cold 20 mM Tris-HCl buffer (Sigma-Aldrich, purity 99%) adjusted to a pH of 7.5 using 1 M NaOH (Sigma-Aldrich, purity 99.99%). After aliquot sampling (1 mL), the homogenate (H) was fractionated by centrifugation at 25,000 g during 1h at 4 °C to separate the insoluble fraction (in the pellet) from the soluble fraction (in the supernatant). Before analyses, samples were mineralized in aqua regia (HNO_3_/HCl, 2:5 v/v, Sigma-Aldrich, purity 99.9%) using a Digiprep block digestion system and filtered at 1 µm. Then, metal(loid) concentrations in the different fractions were obtained by ICP-MS. The certified reference material (TORT-2, lobster hepatopancreas) presented an average recovery efficiency of 89 ± 17%.

### 2.6. Statistical Analyses

Differences in mortality, growth, and metal(loid) concentrations in fractions of *C. riparius* were tested using a F-test (analysis of variance, ANOVA) or a Kruskal-Wallis test (when normality and/or homoscedasticity prerequisites were not fulfilled). Post-hoc pairwise comparisons were then checked using Tukey’s HSD (Honest Significant Difference). Differences were considered statistically significant when *p* < 0.05.

The temporal trends of emergence were described using a non-linear mixed effects regression model (nlme), integrating the station as fixed factor and the cage as random effect. The cumulative emergence percentages were fitted using a logistic model as follows (1):

(1) $$emergence=\frac{A}{1+e^{\left(-kg\left(t-I\right)\right)}}$$

where *A* is the maximal percentage of emergence reached during exposure (%), *kg* the emergence constant (d^−1^), and *I* the time required to reach the inflexion point (d). Significant differences in parameter estimates between stations were checked from the overlap of 95% confidence intervals (CI_95%_).

All statistical analyses were computed using R software (version 4.0.3) using several packages (ggplot2, pgirmess, nlme, lattice, multcomp) [26].

## 3. Results

### 3.1. State of Contamination of the Beuveroux and AMD Identification

The physicochemical characteristics of the Beuveroux, including the dissolved concentrations of metal(loids) are shown in Table 1. It can be noticed an increase of metal(loid) levels in the overlying water from the site entrance (far upstream) to the farthest downstream station. For instance, Cd, Co, Pb and Zn concentrations increased by a factor 60, 28, 9 and 33, respectively. More precisely, this contamination is rapidly quantifiable in the upstream section of the Beuveroux bordering the North part of the heap (station near upstream). It intensified downstream, after the confluence with the resurgence originating from the heap. This tributary, according to its very high metal(loid) concentrations (up to 226 and 27,825 µg.L^−1^ for Cd and Zn, respectively), its high conductivity (655 µS.cm^−1^) and low pH (4.2), can therefore be regarded as an AMD and a punctual source of contamination for the Beuveroux river. Hence, within a few meters after the AMD tributary flows into the river, the concentrations of Cd, Co and Zn rose significantly, as observed at the near downstream station (Table 1). The contamination then persists even several kilometers downstream (far downstream station). Apart from the far upstream station, metal(loid)s concentrations regularly exceed the water quality guidelines (Predicted No Effect Concentrations).

The elementary composition of suspended particles is presented in Table 2 and shows an important and gradual increase of their metal(loid) concentrations along the course of the Beuveroux. The contamination ratios between downstream and upstream stations figure out, for example, that Cd, Ni and Tl concentrations are 8.3, 2 and 1.2 times more important downstream. The measured values exceed the consensual concentrations for a good ecological sediment status [28]. 

### 3.2. Composition of Materials from the Heap

Regarding the levels of metal(loid)s reached in the AMD, their origin has to be sought in the materials constitutive of the heap. 

The mineralogical composition of shale fragments was obtained through XRD analysis and the related diffractograms are presented in Figure S1. Some coarse fragments, as sampled on the West border of the heap, show the presence of quartz (SiO_2_), pyrite (FeS_2_), chlorite ((Fe,Mg,Al)_6_(Si,Al)_4_O_10_(OH)_8_) and muscovite (KAl_2_(AlSi_3_O_10_)(OH,F)_2_). However, in detrital samples, i.e., fine fragments from the former settling pond, chlorite and muscovite, have disappeared. At the same time, goethite (FeO(OH)) and jarosite (K(Fe_3_(SO_4_)_2_(OH)_2_)) were identified (Figure S1). 

**Figure 3:**
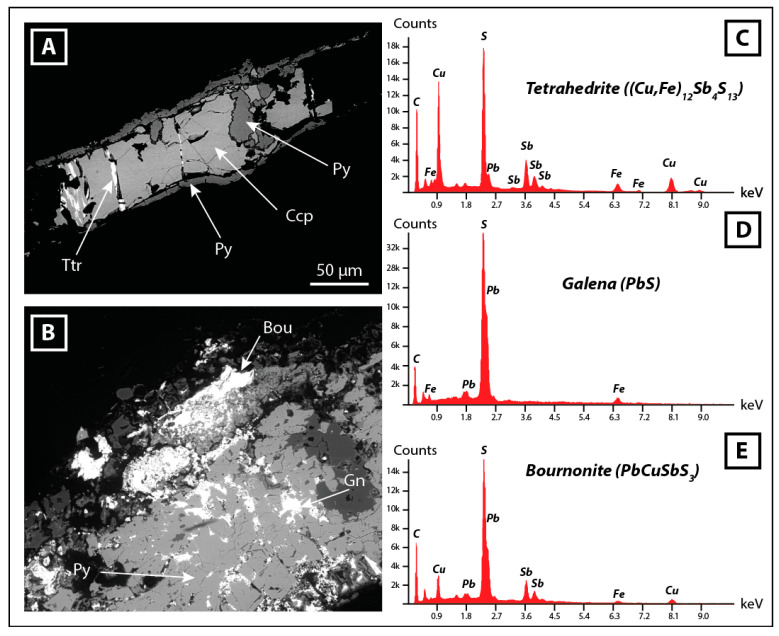
(**A**–**B**): SEM-BSE images of metal bearing sulfides within a shale fragment from the foot of the tailing. Dark grey minerals are silicates (quartz, feldspars, micas) from the shales. (**C**–**E**): EDS spectra of selected sulfide minerals. Abbreviations: Bou: bournonite, Ccp: chalcopyrite, Gn: galena, Py: pyrite, Ttr: tetrahedrite.

XRD analyses were completed by MEB-EDS observation to investigate the potential metal(loid) bearing minerals, which were not revealed by XRD. As expected, pyrite (FeS_2_) is the main mineral phase of the shale residues, but tetrahedrite (Cu_6_[Cu_4_(Fe,Zn)_2_]Sb_4_S_13_), chalcopyrite (CuFeS_2_), galena (PbS) and bournonite (PbCuSbS_3_) were identified as metal-rich minerals (Figure 3). 

Finally, total concentrations of metal(loid)s in materials were chemically quantified and are presented in Table 3. These data corroborate the presence of the main metals (Cu, Fe, Pb, Sb, Zn) constitutive of the minerals. Moreover, quite elevated concentrations of As and Cd were also measured in tailing materials. At the spatial scale of the heap, most of coarse materials present the same metal(loid) concentration pattern. The fine detrital materials of the former settling pond present however the highest concentrations for numerous elements, except for Cd with only 0.5 µg.g^−1^.

### 3.3. Biological Responses of Chironomus Riparius Larvae

#### 3.3.1. Mortality and Growth of Individuals

**Figure 4:**
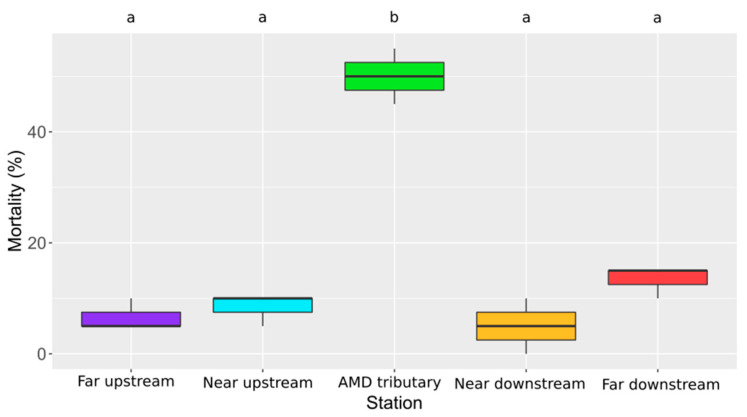
Boxplots of mortality percentage (%) according to the station. Statistical and significant differences between the stations are identified by different letters.

The percentage of mortality in larvae exposed in the different stations is presented in Figure 4. It varies in average between 5% and 13% in the upstream and downstream stations but it reaches 50% in the AMD tributary.

**Figure 5:**
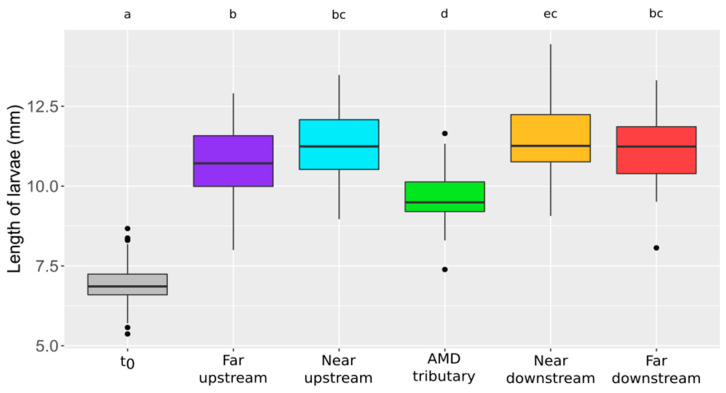
Boxplots of length of larvae (mm) according to the station (30 < *n* < 58). t_0_: beginning of the exposure (*n* = 60). Statistical and significant differences between the stations are identified by different letters.

Concerning growth, the individual lengths of larvae after 7 days of exposure are presented in Figure 5. Individuals have significantly grown up regarding to the length of larvae at the beginning of the exposure (i.e., t_0_). They reached between 10.6 and 11.5 mm in the upstream and downstream stations. In the AMD tributary, a significant growth inhibition could be noticed with larvae measuring only 9.6 mm in average.

#### 3.3.2. Emergence Kinetics

**Figure 6:**
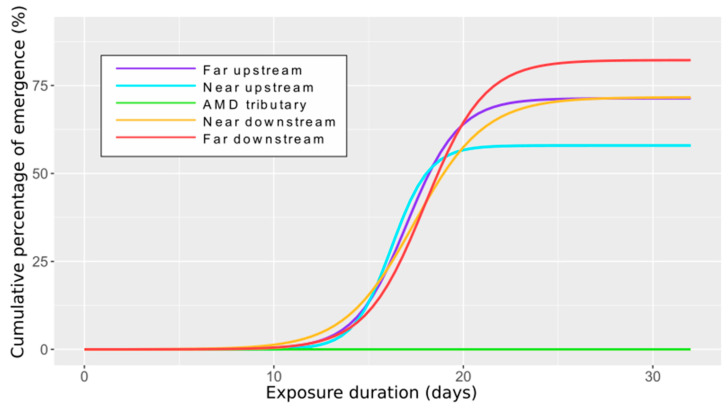
Time course of cumulative percentage of emergence of *C. riparius* encaged at the different stations.

The Figure 6 depicts the emergence kinetics as the cumulative percentage of imagos (i.e., adults) retrieved in the cages over the exposure duration and for the different stations. The modeled patterns are globally comparable between stations located upstream and downstream with a progressive emergence of adults over time. The first emergences were observed after two weeks and the last ones during the fourth week of exposure. In the AMD tributary, no emergence was monitored.

The emergence kinetics can be described according to the modeled parameters presented in Table 4 which permit going deeper in the analysis of the results. For instance, *C. riparius* larvae exposed in the far downstream station showed the highest maximal percentage of adult reached (A = 82.2%) associated to a quite low emergence constant (kg = 0.637 d^−1^) and a delayed achievement of the inflection point (I = 18.0 d). Contrarily, larvae encaged in the near upstream station emerged earlier (I = 16.2 d), faster (kg = 1.001 d^−1^) but with a lower emergence success (A = 57.9%). In the far upstream and near downstream stations, intermediate values and patterns were modeled.

#### 3.3.3. Bioaccumulation of Metals

The bioavailability of metals to *C. riparius* was assessed through the concentrations of metals accumulated in different subcellular compartments, i.e., the soluble and insoluble fractions. The efficiency of fractionation protocol was first assessed thanks to the recovery of metal concentrations between the homogenate and the sum of the soluble and the insoluble fractions. These recoveries reached 103 ± 5% (Table S1) and testify to the reliability of the fractionation protocol.

**Figure 7:**
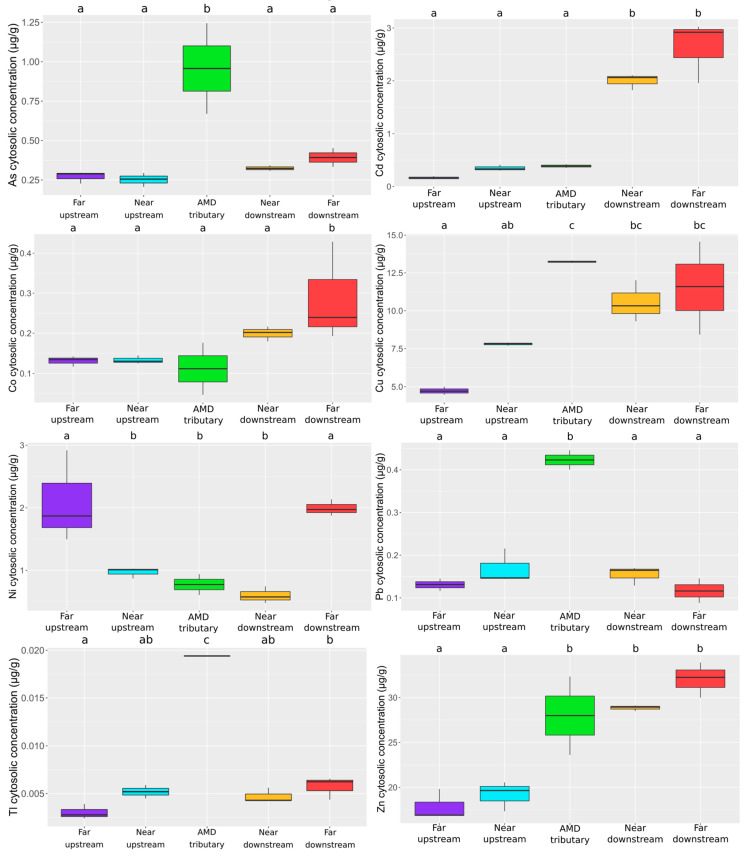
Boxplots of metal accumulation in the cytosolic fraction of *Chironomus riparius* for As, Cd, Co, Cu, Ni, Pb, Tl and Zn (µg.g^−1^, *n* = 3) according to the different stations. Statistical and significant differences between the stations are identified by different letters.

The percentages of contribution between the soluble and insoluble fractions have been calculated for each metal(loid) (Table 5) and show patterns dependent of the element considered. Hence, Cd and Cu were mainly retrieved in the soluble compartment while Al, As, Co, Ni, Pb, Tl and Zn accumulated preferentially, although variably, in the insoluble compartment. A special attention will be paid to the cytosolic (=soluble) fraction regarding its toxicological significance to interpret the biological responses of larvae [16]. According to the metal(loid) and the station considered, we observed contrasted cytosolic bioaccumulation patterns (Figure 7). Hence, concentrations of As, Pb and Tl in the cytosol showed a significant increase (four-fold factor) in larvae exposed in the AMD tributary, while we observed a gradual increase of Cd, Co, Cu and Zn internal concentrations from upstream to downstream stations. Finally, Ni concentrations display a U shape pattern with the highest cytosolic values measured in larvae exposed in far upstream and downstream stations (Figure 7).

## 4. Discussion

### 4.1. From the Elementary and Mineralogical Composition of Materials to Aquatic Compartment Contamination

#### 4.1.1. Origin of the AMD Contamination

The composition of shales in the context of coal mining is well known [29] and we found ubiquist minerals such as quartz, pyrite, chlorite and muscovite. In a shale-water system, the solid-solution chemistry may be affected by numerous interactions and reactions [30]. First, when an oxic fluid comes into contact with the shale residues, these minerals rapidly undergo oxidative dissolution. The study site provided interesting samples illustrating different levels of alteration. Hence, and notably in fine detrital materials from a former settling pond, chlorite and muscovite, easily hydrolysable, were not retrieved any more. Simultaneously, pyrite oxidation generates acidity that could not be completely neutralized by the presence of carbonate-bearing minerals in the shale of the coal basin [20] leading to a pH of the AMD effluent around 4. These acidic conditions then enhance the dissolution of shale tailing materials and the release of water containing high concentrations of dissolved metal(loid)s in the surrounding environment [31,32]. Here, we identified chalcopyrite, galena, tetrahedrite and bournonite as the main source of Cu, Pb, Sb and Zn in the AMD. As some elements may be difficult to detect with spectroscopic tools, complementary chemical analyses also revealed the presence of As, Cd, Co and Ni in the mine tailings. These elements may be part of the composition of minerals such as tetrahedrite but they may also be trapped and concentrated in newly formed phases (e.g., ochre precipitates) [33]. Indeed, in supergene environments, oxidative dissolution of pyrite is commonly accompanied by precipitation of secondary Fe(III)-(oxy)hydroxides [34,35], including jarosite and goethite which were both identified in our samples [36]. All these geochemical processes may explain the elevated concentrations of numerous metal(loid)s in the fine detrital materials from the former settling pond. However, these wastes contained only small amounts of Cd, probably in relation with the high solubility and mobility of this element, especially under acidic conditions [37]. 

Whether the metal(loid)s originate from primary or secondary mineral phases or products, they may rapidly reach, through rainfall events, adjacent water bodies under particulate (flushing of the accumulated oxidation products) or dissolved forms.

#### 4.1.2. Contamination of the AMD Receiving Waters

The contamination of the water and suspended particles far upstream in the Beuveroux river (i.e., before the river reaches the tailings) was relatively low and representative of the local geological and geochemical background (coal basin; [38]). From the near upstream station, these dissolved and particulate metal(loid)s concentrations increased drastically. This could be attributable to the diffuse leaching of tailings by percolating rainfall over the considerable surface of the heap. Moreover, the drainage of contaminated effluents may be facilitated in the north part of the heap by the presence of the former riverbed of the Beuveroux which have been covered during tailing reworking in the 80’s. A punctual source of contamination was also clearly identified to the west border of the heap (AMD tributary). Regarding its extremely high sulfur and metal(loid) levels and low pH, this tributary meets the definition of an AMD. From its confluence with the Beuveroux river, the contamination continues to increase and persists even a few kilometers downstream from the site. There, although the legacy mining wastes are the main contributors to this contamination, other sources originating from the industrial and agricultural activities in the watershed may also be involved. 

Hence, once the Beuveroux river begins flowing along the heap, the concentrations measured in water and suspended particles exceed the environmental quality guidelines for all the metal(loid)s investigated [27,28]. This clearly evidences a chemical pollution and raises the question of the potential impacts on biotic compartments.

### 4.2. Water Quality and Its Implications for the Biotic Compartment

#### 4.2.1. Ecotoxicity of AMD and the Beuveroux

The ecotoxicity of the AMD and the receiving water of the Beuveroux was assessed through different biological endpoints: the survival of *C. riparius* larvae, but also, because lethal endpoints can be less sensitive than sub-lethal ones, their growth and the emergence of adults [13,39]. In the far downstream and upstream stations, we did not observe any toxic effects. The survival (87–93%) and emergence (71–82%) rates were similar to those observed in situ for uncontaminated sites (e.g., 77–87% and 84–88%, respectively; [14]). In the near downstream and upstream stations, only quite limited effects were monitored on the emergence. In the AMD tributary, mortality and growth inhibition were already elevated after only seven days of exposure. After one month of exposure, all the larvae were dead explaining the absence of emergence in this station. These contrasted responses according to the station may be related to the concomitant influence of different factors. Firstly, the acidic conditions of the AMD may be a factor of stress, although larvae of genus *Chironomus* are quite resistant to low water pH [40]. Secondly, the food availability, less important in the AMD tributary lacking of suspended particles compared to the other stations in the Beuveroux river, may represent a limiting factor for survival and growth [41]. This constrain may be of peculiar importance considering that *C. riparius* larvae are all the more tolerant to metallic contamination as food is provided in sufficient quantity and quality [42]. Hence, in the far downstream station, the quantity of suspended particles may have balanced the exposure to relatively high concentrations of metal(loid)s. In the AMD tributary, the situation is reversed. The intense exposure to metal(loid)s may lead to modifications in the energetic allocation between somatic maintenance (excretion and/or detoxification processes), and growth/emergence [43]. In this sense, further investigations regarding the fate of accumulated metal(loid)s in larvae tissues could help to better understand the origin of the toxic responses observed.

#### 4.2.2. Internal Distribution and Toxicological Bioavailability of Metal(loid)s to C. Riparius

Metal fate, both in abiotic and biotic compartments of freshwater ecosystems, is a relevant issue especially for environmental quality assessment. In the context of AMD, the availability of metal(loid)s is often complex to determine due to particular physico-chemical conditions (pH, oxidation–reduction potential, presence of numerous dissolved species…) affecting their partitioning, speciation and mobility in aqueous environments [32,44]. Moreover, the bioavailability, bioaccumulation and toxicity of metal(loid)s depend on the target organism considered and its physiological features controlling the internal distribution of the contaminants. Hence, assessing the toxicological bioavailability and the risk associated to metal mixtures will particularly benefit from the monitoring of accumulation in subcellular compartments, especially the cytosol [15,16,24,25].

Our results allowed to distinguish three bioaccumulation patterns regarding the concentrations of the different metal(loid)s in the cytosolic fraction. Firstly, Ni appears less toxicologically bioavailable in the AMD effluent compared to the other stations. This is in accordance with previous studies showing that accumulated Ni is mainly sequestered in the insoluble fraction (probably in granules) in exposed *C. riparius* [15,45]. Secondly, Cd, Co, Cu and Zn showed increasing cytosolic concentrations from upstream to downstream stations in correlation with the suspended particles contamination. Copper and Zn present a high affinity for suspended organic matter [46]. As they serve as food item for *C. riparius* larvae [15,47], suspended particles represent probably the main contamination source for these elements. However, once assimilated, the internal fate of these metals is not the same. Cobalt and Zn may be excreted by aquatic invertebrates [48,49] and are mainly retrieved in the insoluble fraction. On the contrary, Cd and Cu accumulate in the cytosolic fraction where they are durably bound notably to metallothionein (MT), proteins implicated in their detoxification and sequestration [15,50,51]. Finally, As, Pb and Tl were particularly accumulated by larvae exposed to the AMD which testifies to their environmental and toxicological bioavailability. Due to its affinity for sulfur-rich ligands [52], half of the accumulated Tl is sequestered in the cytosolic compartment in larvae exposed to the AMD effluent. Probably under a detoxified form, its toxic potential is quite limited and *C. riparius* larvae are tolerant to Tl [53]. Moreover, the concentrations reached were quite low (around 0.02 µg.g^−1^) probably in relation with the larvae ability to efficiently excrete this element through the Malpighian tubules [54]. On the other hand, As and Pb were mainly accumulated in the insoluble fraction but their levels in soluble sensitive compartments were four times higher than in other stations. These concentrations in such a sensitive compartment may have been sufficient to interact with sites of toxic action (e.g., enzymes), disrupt biological functions and cause the effects observed [55,56,57].

Since chironomid larvae represent an important feeding resource for numerous aquatic (macroinvertebrates, fishes) and terrestrial (birds) species, knowledge of the internal distribution of the accumulated meal(oid)s is a valuable tool for interpreting their potential ecotoxicological significance for higher trophic level. Some studies show that the contaminants stored in the cytosolic fraction may be trophically available for some predators, according to the strength of their digestive processes [25]. Here, it represented up to 60, 45, and 20%, respectively, of the total Cd, Cu, and Co or Zn concentrations in the exposed larvae.

### 4.3. Mitigation and Remediation Perspectives

The AMD phenomenon identified led to a significant environmental hazard and risk that need to be addressed by mitigation and/or remediation measures. 

First, it could be envisaged to intervene directly on the source of the observed contaminations, i.e., the mining wastes. However, the complete re-exploitation or removal of the metal-rich tailings constitutive of the heap are largely unsustainable, considering the amount of materials concerned and the associated costs. As AMD effluents originate in the interaction between the shale residues, atmospheric oxygen and water, the control of water infiltration in the heap could however be a relevant lever. From a hydrogeological point of view, we identified two potential point sources of water inputs: a little stream coming from a former mining shaft (Puits Arthur) infiltrating through the South-West face of the heap and the Beuveroux itself through its former riverbed now covered by the northern part of the heap. Although drainage works may be implemented to deviate these two point sources, it is however not possible to waterproof the entire surface of the heap in order to avoid the percolation of rainfall, which contributes significantly to the diffuse leaching of mining wastes.

Secondly, the AMD effluent itself can be managed using active or passive treatments developed to raise pH and remove metals. Active technologies include chemical treatments such as adsorption process, selective precipitation, membrane technology and electrochemical process but also biological treatments using bacteria or microalgae in bioreactors [58]. Passive approaches implement flow-through systems bringing into contact the AMD effluent with various substrates, including inert (e.g., siderurgical slags were proposed as an efficient low-cost material [59]) or organic (e.g., compost) matrices. Phytoextraction and phytostabilization processes using different algae or plant species are also applied for AMD remediation [60,61]. Although these technologies may be limited regarding their efficiencies in metal removals in comparison to chemical treatments, their ecological relevance and cost-effectiveness could be compatible with the financial and human resources allocable by site owners or managers. 

Finally, although AMD remediation may remain a challenge especially for small municipalities often financially constrained, it may also represent a source of valuable resources. Indeed, the combined implementation of complementary treatments can allow the reuse of cleaned water, the production of biomass and the recovery of metals which improve the cost-effectiveness and the applicability of such mitigation and remediation technologies.

## 5. Conclusions

The generation of acidic drainage and the release of water containing high concentrations of dissolved metal(loid)s from mining wastes represent an environmental problem worldwide. Through an interdisciplinary study, we were able to identify the mineralogical origin of the contamination from legacy coal mine tailings, its fate in receiving waters and its ecotoxicity to aquatic invertebrates. The weathering of metal(loid)-bearing minerals (such as bournonite and tetrahedrite) leads to the release of numerous metal(loid)s in diffuse and punctual AMD effluents. Once discharged into the river bordering the heap, some metals (e.g., Cd, Co, Zn) may diffuse at large scale through their association with suspended particles. Thanks to original biomonitoring devices, we were able to characterize the toxicological bioavailability of numerous metal(loid)s to *C. riparius* larvae. Their survival, growth and emergence were disrupted to varying degrees and subcellular fractionation analyses pointed out the implication of notably As and Pb in the toxic responses. We therefore clearly evidenced that several decades after the cessation of mining operation, the shale tailings still constitute a chronic and diffuse source of metal(loid) contamination and require the implementation of management policies. 

## Figures and Tables

**Table 1:** Physicochemical characteristics (pH and conductivity in µS.cm^−1^) and metal concentrations (µg.L^−1^) in the Beuveroux river, upstream and downstream to the acid mine drainage (AMD) tributary. Data are mean ± standard deviation (*n* = 3). DL: detection limit. PNEC: Predicted No Effect Concentrations in water [27].

Station	Al	As	Cd	Co	Cu	Ni	Pb	Tl	Zn	pH	Conducti-vity
Far upstream	51.8 ± 21.4	5.52 ± 0.10	0.08 ± 0.02	0.23 ± 0.00	<DL	0.90 ± 0.06	0.28 ± 0.04	0.01 ± 0.00	41.0 ± 10.7	7.28 ± 0.03	116 ± 13
Near usptream	90.7 ± 24.8	9.19 ± 5.40	0.51 ± 0.61	0.33	14.6	3.28 ± 3.16	0.77 ± 0.64	0.04 ± 0.04	205 ± 217	7.19 ± 0.11	122 ± 18
AMD Tributary	15,585 ± 6611	1.31 ± 0.81	226 ± 185	158 ± 25	116 ± 48	270 ± 119	109 ± 82	0.81 ± 0.70	27,825 ± 12,551	4.20 ± 0.06	655 ± 12
Near downstream	107 ± 50	4.71 ± 0.30	0.96 ± 0.32	1.68 ± 0.63	4.62	3.11 ± 0.79	0.60 ± 0.20	0.02 ± 0.00	344 ± 110	6.76 ± 0.01	157 ± 47
Far downstream	211 ± 20	12.4 ± 4.3	4.79 ± 2.21	6.49 ± 2.93	12.7 ± 12.9	11.9 ± 4.3	2.46 ± 0.36	0.08 ± 0.01	1349 ± 555	7.29 ± 0.00	126 ± 19
PNEC		4.2	0.08		1.4	3.8	2.3		10.8		

**Table 2:** Metal concentrations in suspended particles (µg.g^−1^) collected at the different stations.

Station	Al	As	Cd	Co	Cu	Ni	Pb	Tl	Zn
Far upstream	13,906	50.7	3.31	9.34	36.6	17.9	55.8	0.51	747
Near upstream	17,610	84.8	5.30	14.6	38.5	22.0	63.4	0.56	1110
Near downstream	17,665	84.5	8.92	17.9	40.5	23.9	53.7	0.55	1784
Far downstream	21,044	129.6	28.4	42.4	67.0	35.9	71.9	0.62	3457
Ratio far downstream/far upstream	1.5	2.6	8.3	4.5	1.8	2.0	1.3	1.2	4.5
Reference values *		7.90	0.93		14.0	11.0	25.0		146

* Consensus values for a good ecological sediment status [28].

**Table 3:** Metal concentrations (µg.g^−1^) in constitutive materials of the tailing according to the different sampling points.

Sampling Point	Al	As	Cd	Co	Cu	Fe	Ni	Pb	S	Sb	Zn
Former settling pond	51,142	627.7	0.50	18.1	200.3	69,809	27.5	3223.7	9064	140.9	1267.0
Proximity to AMD	46,544	727.8	6.87	19.1	47.8	50,041	28.5	424.7	5377	40.00	990.1
East border of the tailing	48,032	661.8	3.48	13.4	44.5	37,524	17.1	424.9	4217	28.04	300.5
Foot of the tailing	25,400	11.6	3.35	7.2	34.5	12,660	20.8	269.4	3995	19.60	383.2
West border of the tailing	42,086	214.3	1.12	12.6	38.5	25,440	21.7	262.5	3085	18.86	258.7

**Table 4:** Kinetic parameters of emergence modelled for stations (mean and 95% confidence intervals). A is the maximal percentage reached (%), kg the emergence constant (d^−1^) and I the time at the inflection point (d). Different letters indicate significant differences between stations.

Station	A (%)			Kg (d^−1^)			I (d)		
	Lower Limit	Estimated Value	Upper Limit	Lower Limit	Estimated Value	Upper Limit	LOWER Limit	Estimated Value	Upper Limit
Far upstream	64.1	71.4 ^ab^	78.6	0.352	0.728 ^ab^	1.104	15.7	17.0 ^ab^	18.4
Near upstream	51.6	57.9 ^a^	64.3	0.840	1.001 ^a^	1.162	15.6	16.2 ^a^	16.8
AMD Tributary	-	-	-	-	-	-	-	-	-
Near downstream	67.7	71.7 ^b^	75.6	0.374	0.539 ^b^	0.704	16.6	17.4 ^ab^	18.2
Far downstream	78.8	82.2 ^c^	85.6	0.502	0.637 ^b^	0.772	17.7	18.0 ^b^	18.2

**Table 5:** Contribution (%) of the soluble and the insoluble fractions in bioaccumulation of metals in *C. riparius* larvae. Data are mean ± standard deviation (*n* = 3). Different letters indicate significant differences between stations.

Station	Fraction	Al	As	Cd	Co	Cu	Ni	Pb	Tl	Zn
Near downstream	Soluble	2.0 ± 0.3	16.3 ± 0.4	63.8 ± 0.3	28.7 ± 3.6	61.6 ± 1.8	25.3 ± 3.7	6.1 ± 1.0	20.0 ± 3.8	37.8 ± 1.7
AMD Tributary		2.3 ± 0.1	5.4 ± 0.9	59.6 ± 3.5	23.1 ± 14.0	45.3 ± 3.6	5.5 ± 0.6	3.6 ± 0.6	44.9 ± 6.1	19.6 ± 2.2
Near downstream	Insoluble	98.0 ± 0.3	83.7 ± 0.4	36.2 ± 0.3	71.3 ± 3.6	38.4 ± 1.8	74.7 ± 3.7	93.9 ± 1.0	80.0 ± 3.8	62.2 ± 1.7
AMD Tributary		97.7 ± 0.1	94.6 ± 0.9	40.5 ± 3.5	76.9 ± 14.0	54.7 ± 3.6	94.6 ± 0.6	96.4 ± 0.5	55.1 ± 6.1	80.4 ± 2.2

## Supplementary Materials

The following are available online at https://www.mdpi.com/article/10.3390/toxics9070164/s1, Figure S1: “XRD patterns of bulk powders of shale from the studied area (A: West border of the tailing, B: former settling pond and C: proximity to AMD). Abbreviations Chl: chlorite, Ms: muscovite, Qz: quartz, Py: pyrite, Gth: goethite and Jrs: jarosite”, Table S1: “Concentrations of metal(loid)s bioaccumulated in the homogenate (H), soluble (S) and insoluble (P) fractions with a comparative relative difference (%) between H fraction and the sum of S and P”.
