# Supplementary material for: A Multidisciplinary Approach for the Assessment of Origin, Fate and Ecotoxicity of Metal(loid)s from Legacy Coal Mine Tailings
Source: Toxics. 2021 Jul 10;9(7):164. doi: 10.3390/toxics9070164 (PMC8309815; doi:10.3390/toxics9070164)
Supplement: Supplementary file 1 [file toxics-09-00164-s001.zip › toxics-1261821-supplementary.pdf]

toxics

Article

Supplementary Materials: A Multidisciplinary Approach for the Assessment of Origin, Fate and Ecotoxicity of Metal(loid)s from Legacy coal Mine Tailings

Honorine Gauthier-Manuel, Diane Radola, Flavien Choulet, Martine Buatier, Raphaël Vauthier, Tatiana Morvan, Walter Chavanne and Frédéric Gimbert

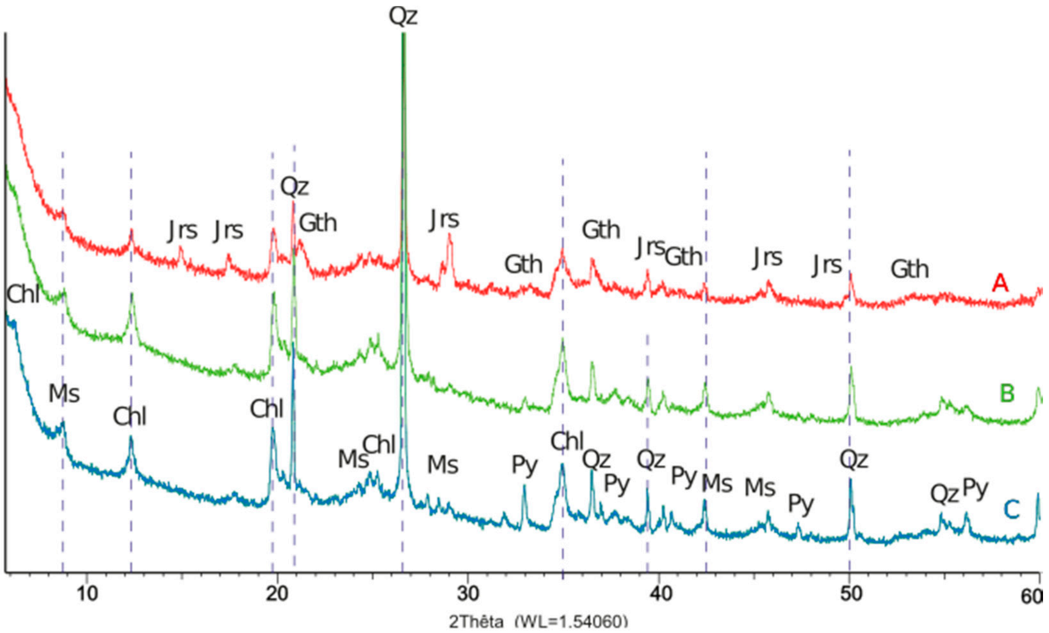

Figure S1. XRD patterns of bulk powders of shale from the studied area (A: West border of the tailing, B: former settling pond and C : proximity to AMD). Abbreviations: Chl: chlorite, Ms: muscovite, Qz: quartz, Py: pyrite, Gth: goethite and Jrs: jarosite.

Table S1. Concentrations of metal(loid)s bioaccumulated in the homogenate (H), soluble (S) and insoluble (P) fractions with a comparative relative difference (%) between H fraction and the sum of S and P.

Fraction	Station	Al	As	Cd	Co	Cu	Ni	Pb	Tl	Zn
H	Near downstream 1	93.3	2.04	2.92	0.66	13.9	3.77	2.57	0.023	79.8
H	Near downstream 2	81.3	1.76	2.51	0.71	13.3	2.21	2.21	0.018	79.3
H	Near downstream 3	87.4	1.91	3.21	0.66	18.7	1.66	2.65	0.022	73.7
H	Tributary 1	509.0	13.6	0.79	0.63	27.9	17.2	11.2	0.04	155.8
H	Tributary 2	724.8	22.0	0.72	0.35	33.0	10.6	12.9	0.05	138.9
S+P	Near downstream 1	114.7	2.16	3.23	0.73	15.6	3.37	2.56	0.025	80.6
S+P	Near downstream 2	100.6	1.96	2.87	0.71	16.3	2.35	2.49	0.023	75.2
S+P	Near downstream 3	89.0	1.85	3.30	0.65	19.4	1.63	2.55	0.023	73.4
S+P	Tributary 1	553.3	14.1	0.68	0.53	27.5	18.7	11.2	0.04	152.8
S+P	Tributary 2	705.7	20.6	0.62	0.35	31.1	10.4	12.5	0.05	130.5
Relative difference between H and S+P (%)	Near downstream 1	122.9	105.8	110.0	110.9	112.0	89.6	99.5	110.8	101.1
	Near downstream 2	123.7	111.3	114.7	100.0	122.4	106.1	112.6	128.7	94.9
	Near downstream 3	101.8	96.9	102.7	99.0	103.9	98.0	96.4	106.1	99.5
	Tributary 1	108.7	103.9	86.2	84.8	98.5	108.5	100.1	103.9	98.1
	Tributary 2	97.4	93.5	85.4	101.0	94.4	97.4	97.3	97.1	93.9
	Mean	110.9	102.3	99.8	99.1	106.2	99.9	101.2	109.3	97.5
	SD	12.0	7.1	13.5	9.3	11.2	7.6	6.6	11.9	3.0
